# Genome sequence of the stramenopile *Blastocystis*, a human anaerobic parasite

**DOI:** 10.1186/gb-2011-12-3-r29

**Published:** 2011-03-25

**Authors:** France Denoeud, Michaël Roussel, Benjamin Noel, Ivan Wawrzyniak, Corinne Da Silva, Marie Diogon, Eric Viscogliosi, Céline Brochier-Armanet, Arnaud Couloux, Julie Poulain, Béatrice Segurens, Véronique Anthouard, Catherine Texier, Nicolas Blot, Philippe Poirier, Geok Choo Ng, Kevin SW Tan, François Artiguenave, Olivier Jaillon, Jean-Marc Aury, Frédéric Delbac, Patrick Wincker, Christian P Vivarès, Hicham El Alaoui

**Affiliations:** 1 Genoscope (CEA) and CNRS UMR 8030, Université d'Evry, 2 rue Gaston Crémieux, 91057 Evry, France; 2 Clermont Université, Université Blaise Pascal, Laboratoire Microorganismes: Génome et Environnement, BP 10448, F-63000 Clermont-Ferrand, France; 3 CNRS, UMR 6023, LMGE, F-63177 Aubière, France; 4 Center for Infection and Immunity of Lille, Institut Pasteur de Lille, F-59019 Lille Cedex, France; 5 Inserm U1019, F-59000 Lille Cedex, France; 6 CNRS UMR 8402, F-59021 Lille Cedex, France; 7 University Lille-Nord de France, F-59000 Lille Cedex, France; 8 Laboratoire de chimie bactérienne (CNRS UPR9043), Institut de Microbiologie de la Méditerrannée, 31 chemin Joseph Aiguier, 13402 Marseille, France; 9 Université de Provence, Aix-Marseille I, 3 place Victor Hugo, 13331 Marseille, France; 10 Laboratory of Molecular and Cellular Parasitology, Department of Microbiology, Yong Loo Lin School of Medicine, National University of Singapore, Singapore, 5 Science Drive 2, 117597 Singapore

## Abstract

**Background:**

*Blastocystis *is a highly prevalent anaerobic eukaryotic parasite of humans and animals that is associated with various gastrointestinal and extraintestinal disorders. Epidemiological studies have identified different subtypes but no one subtype has been definitively correlated with disease.

**Results:**

Here we report the 18.8 Mb genome sequence of a *Blastocystis *subtype 7 isolate, which is the smallest stramenopile genome sequenced to date. The genome is highly compact and contains intriguing rearrangements. Comparisons with other available stramenopile genomes (plant pathogenic oomycete and diatom genomes) revealed effector proteins potentially involved in the adaptation to the intestinal environment, which were likely acquired via horizontal gene transfer. Moreover, *Blastocystis *living in anaerobic conditions harbors mitochondria-like organelles. An incomplete oxidative phosphorylation chain, a partial Krebs cycle, amino acid and fatty acid metabolisms and an iron-sulfur cluster assembly are all predicted to occur in these organelles. Predicted secretory proteins possess putative activities that may alter host physiology, such as proteases, protease-inhibitors, immunophilins and glycosyltransferases. This parasite also possesses the enzymatic machinery to tolerate oxidative bursts resulting from its own metabolism or induced by the host immune system.

**Conclusions:**

This study provides insights into the genome architecture of this unusual stramenopile. It also proposes candidate genes with which to study the physiopathology of this parasite and thus may lead to further investigations into *Blastocystis*-host interactions.

## Background

*Blastocystis *sp. is one of the most frequent unicellular eukaryotes found in the intestinal tract of humans and various animals [1]. This anaerobic parasite was first described by Alexeieff at the beginning of the 20th century [2]. For a long time, the taxonomy of *Blastocystis *was controversial. Despite the application of molecular phylogenetic approaches, it was only recently that *Blastocystis *sp. was unambiguously classified within the stramenopiles [3-5]. This eukaryotic major lineage, also called Heterokonta, encompasses very diverse organisms (unicellular or multicellular, heterotrophic or photosynthetic) such as slime nets, diatoms, water moulds and brown algae [6]. One important characteristic of stramenopiles is the presence during the life cycle of a stage with at least one flagellum permitting motility. It is important to note that *Blastocystis *sp. does not possess any flagellum and is the only stramenopile known to cause infections in humans [4]. For the organism isolated from human fecal material, Brumpt suggested the name *Blastocystis hominis *[7]. However, as the species *B. hominis *is difficult to establish, we use the term '*Blastocystis *sp.' to designate any subtype observed in humans. *Blastocystis *sp. is the most frequent protozoa reported in human fecal samples [8], with a worldwide distribution [9-13] and a prevalence ranging between 30 and 60% in some developing countries [1]. In addition, infection with *Blastocystis *sp. appears to be common and more severe in immunocompromised or hemophilic patients [9,14,15]. The presence of *Blastocystis *representatives has also been reported in a variety of mammals, birds, reptiles, and even insects [16-18]. *Blastocystis *sp. exhibits extensive genetic diversity, and on the basis of molecular analysis of the small subunit RNA gene, ten distinct subtypes (ST1 to ST10) have been identified from primates (including humans), other mammals and birds [17]. Some arguments support zoonotic transmission to humans, including the high prevalence of ST1 to ST3 in humans and other mammals [17] and the experimental transmission of different human genotypes to chickens, rats and mice [19,20].

The life cycle of *Blastocystis *sp. remains elusive, although different morphological forms have been described, including vacuolar, granular, amoeboid and cysts. Recently, Tan [1] suggested a life cycle with the cyst as the infectious stage. After ingestion of cysts, the parasite may undergo excystation in the gastrointestinal tract and may develop into a vacuolar form that divides by binary fission. The following stage could be either the amoeboid form or the granular form. Then, encystation may occur during passage along the colon before cyst excretion in the feces. Therefore, *Blastocystis *sp. lives in oxygen-poor environments and is characterized by the presence of some double-membrane surrounded-organelles showing elongate, branched, and hooked cristae [21] called mitochondria-like organelles (MLOs) [22]. These cellular compartments contain a circular DNA molecule and have metabolic properties of both aerobic and anaerobic mitochondria [23,24].

*Blastocystis *sp. has been reported as a parasite causing gastro- and extra-intestinal diseases with additional persistent rashes, but a clear link of subtypes to the symptomatology is not well established [11]. Other studies have shown that the parasite can be associated with irritable bowel syndrome [20,25] or inflammatory bowel disease [26]. Thus, the pathogenic role of *Blastocystis *sp. as the primary cause of enteric symptoms is dubious. Therefore, it is important to search for other molecular markers for an epidemiologically integrated study [17]. Here we report the complete genome sequence of a subtype 7 isolate from a Singaporean patient [GenBank:CABX01000000]. Its comparison with the two other available stramenopile genome sequences (that is, *Phytophthora sojae*, a plant pathogenic oomycete, and *Thalassiosira pseudonana*, a free diatom) allows us to highlight some genome-specific features of *Blastocystis *to understand how this parasite evolved within environmental constraints, but also provides a better knowledge of its metabolic and physiological capacities, such as the functioning and the role of MLOs and the arsenal produced to interact or to counter immune defense systems of its host.

## Results and discussion

### General features of the *Blastocystis *genome

The genome of a *Blastocystis *subtype 7 was resolved by pulsed-field gel electrophoresis, and 15 chromosomic bands have been characterized. The final assembled sequence is distributed in 54 scaffolds and the deduced genome is 18.8 Mb in size (16.5-fold sequence coverage), which is much smaller than plant parasite stramenopiles (*Phytophthora infestans*, 240 Mb; *P. sojae*, 95 Mb; *Phytophthora ramorum*, 65 Mb) and also smaller than free stramenopiles (*Phaeodactylum tricornutum*, 27.4 Mb; *T. pseudonana*, 34.5 Mb). The reference annotation of the *Blastocystis *subtype 7 genome contains 6,020 genes, covering about 42% of the genome (Table 1). The average number of exons per gene is 4.6 for multiexonic genes and 929 genes are monoexonic. Compaction in this parasite genome is reflected by the short length of the intergenic regions (1,801 bp), the relatively low repeat coverage (25%) and, more strikingly, by the very short size of introns, with a sharp length distribution of around 32 nucleotides (Figure S1 in Additional file 1). A total of 38 rDNA units organized in transcriptional units, including a small subunit rRNA gene, a 5.8S rRNA gene, and a large subunit rRNA gene in a 5'-3' orientation, have been detected in the genome. The sizes of the small subunit, the large subunit and the 5.8S rRNA gene are 1.8 kb, 2.45 kb and 0.44 kb, respectively. Some units are tandemly duplicated, up to four copies on scaffold 18, and some may also be localized in subtelomeric regions, as revealed by a co-mapping of telomeric sequences and rDNA subunits at scaffold 6 and 9 extremities. These two scaffolds could correspond to entire chromosomes. Due to the sequencing method, some units are incomplete (either truncated or lacking genes). The alignment of 20 complete small subunit rRNA genes shows polymorphism between copies, which is also the case for 29 large subunit rRNA gene copies.

**Table 1:** General features of *Blastocystis *sp. subtype 7

	Number	Mean length	Median length	Total length (Mb)	Percentage of genome (18.8 Mb)
Genes	6,020	1,299	1,397	7.82	42%
Exons	24,580	280	150	6.88	37%
Introns	18,560	50.5	31	0.94	5%
Intergenic	-	1,801	4,092	10.9	58%
Repeats	2,730	1,747	2,862	4.8	25%

The number of genes in *Blastocystis *(6,020) is reduced in comparison with other stramenopiles (*P. infestans*, 17,797; *P. sojae*, 19,027; *P. ramorum*, 15,743; *P. tricornutum*, 10,402; *T. pseudonana*, 11,776). Surprisingly, a large portion of genes were probably duplicated since 404 clusters of paralogous protein-coding genes were identified, containing 1,141 genes, that is, 19% of *Blastocystis *genes (see Material and methods). Excluding the large multigenic families (up to 32 genes with a histone-fold domain and 20 genes with a 4Fe-4S ferredoxin domain), most of the duplicated genes are present in only two copies (Figure S2 in Additional file 1). As described in other organisms [27,28], the duplicated genes are more conserved than single copy genes in *Blastocystis *sp. Indeed, they have more orthologs (defined as best reciprocal hit (BRH); see Materials and methods) and display higher similarities with their orthologs (Figure S3 in Additional file 1). They also tend to display higher expression levels than single copy genes (Figure S4 in Additional file 1).

**Figure 1:**
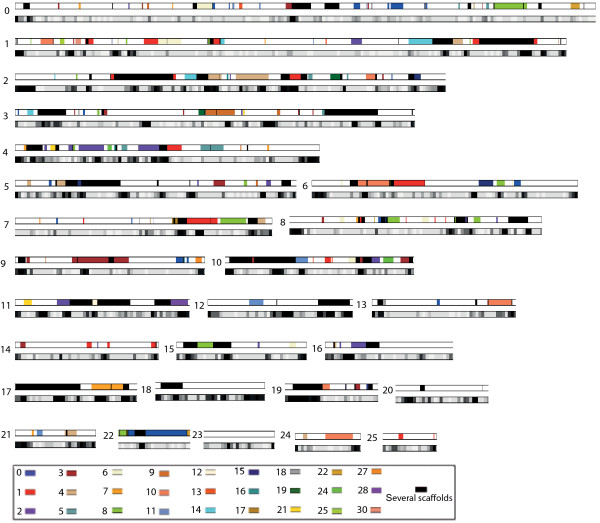
**Blocks of duplicated genes in the *Blastocystis *sp. genome**. For each scaffold (from 0 to 25), the duplicated blocks are displayed with colors corresponding to the scaffolds where the paralogous blocks are located (on scaffolds 0 to 19, 21, 22, 24, 25, 27, 28, 30). Below each scaffold, the repeat density is displayed as a grey scale: 0% (white) to 100% (black) repeats in 10-kb windows.

We investigated whether these gene duplications could have arisen from a whole genome duplication (WGD) or smaller scale segmental duplications. WGD, the duplication of the entire genome by polyploidization, has been shown to have played a key role in the evolutionary history of several animal and plant lineages [27,29-31]. Segmental duplications occur continually by several mechanisms that can duplicate parts of genes, entire genes, or several adjacent genes. These mechanisms include unequal crossing over, or gene conversion, and tandem duplication [32-34]. We were able to identify 320 blocks of duplicated genes, that is, paralogous segments of several adjacent genes (see Materials and methods), some of which are very large (up to 100 kb), suggesting a WGD. These blocks cover about 39% of the genome (7.3 out of 18.8 Mb) representing 38% (5.15 out of 13.65 Mb) of the unrepeated fraction of the genome. As shown in Figure 1, each scaffold is a mosaic of blocks of homology with several other scaffolds: scaffolds cannot be grouped by pairs as would be expected from a recent WGD. Additionally, some segments are present in more than two copies in the genome (they appear in black in Figure 1), suggesting that segmental duplications are likely to have played a role in the current duplication pattern. However, the duplicated blocks are not often on the same scaffold, nor in tandem, which rules out the tandem duplication model. The comparison of paralogous copies shows surprisingly high nucleic acid identity rates: on average, 99% in coding regions, 98.4% in untranslated regions, and 97.8% in introns and intergenic regions. Interestingly, those values are homogeneous among all paralogous blocks, suggesting that all blocks were duplicated at the same time.

Two hypotheses could explain the origin of these duplicated blocks. First, the duplicates may have arisen from a whole genome duplication that took place recently (since the copies are still very similar) and was followed by rapid genome rearrangements and losses of gene copies. The high homology between gene copies could also result from a high rate of homogenization through gene conversion driven by the high frequency of rearrangements. The frequent rearrangements in the *Blastocystis *lineage are probably also the reason why no extensive synteny could be detected between *Blastocystis *sp. and other stramenopiles. Second, the duplicates could also have occurred through segmental duplications (favored by the high rate of rearrangements), although the relatively uniform divergence between copies is more symptomatic of a single event and would imply a burst of segmental duplications during a short period or a very high rate of homogenization by recombination. The intriguing pattern of gene duplications, likely caused by the high rate of rearrangements in the *Blastocystis *genome, makes it impossible to determine which scenario is the most likely. It could be interesting to sequence other subtypes to determine whether the high rate of recombination (loss of synteny) and the pattern of duplications observed in subtype 7 is a common feature within this lineage.

### Endosymbiotic and horizontal gene transfers in *Blastocystis *sp

Phylogenetic analyses revealed two genes of possible cyanobacterial origin in the genome of *Blastocystis*, those encoding phosphoglycerate kinase [GenBank:CBK20833] and 6-phosphogluconate dehydrogenase [GenBank:CBK22626] (Figure S5 in Additional file 1). It is important to notice that 6-phosphogluconate dehydrogenase-encoding genes have been identified in non-photosynthetic protists such as Heterolobosea (not shown). This was interpreted as secondary horizontal gene transfer (HGT) from photosynthetic eukaryotes to Heterolobosea [35,36].

The presence of plastids in various photosynthetic stramenopile lineages (for example, diatoms, chrysophytes, raphidophytes) was interpreted as a secondary endosymbiosis that occurred between a red algae and the ancestor of these groups. By contrast, the evolutionary meaning of the lack of plastids in some heterotrophic stramenopile lineages (for example, oomycetes, bicosoesids) is still under discussion: does it indicate secondary losses of the plastid acquired by the ancestor of all stramenopiles? Or does it reflect the fact that the secondary endosymbiosis at the origin of stramenopile plastids did not occur in their common ancestor but after the divergence of heterotrophic lineages [37]? The presence of genes of cyanobacterial origin in *Blastocystis *supports the first hypothesis even if we can not rule out possible recent acquisitions of genes of chloroplastic origin from photosynthetic eukaryotes as in the case of Heterolobosea.

HGT is important in evolution as an adaptive mechanism of microbial eukaryotes to environmental conditions [38,39] and is known to play an important role in stramenopiles. For instance, iron is a limiting nutrient in surface waters for diatoms. Therefore, the likely acquisition of ferritin by HGT from bacteria has permitted some species to acquire this nutrient from the environment [40]. This is also the case for the diatom *Phaeodactylum*, in which nitrogen metabolism, cell wall silification, DNA replication, genome repair and recombination processes have been shaped by HGT [40,41]. HGT seems also to play an important role in oomycetes since it may be involved in osmotrophy. Genes involved in absorbing products of degradation of complex nutrients were predicted to be candidates for fungi-to-oomycete HGT [42]. By analyzing the set of predicted genes in *Blastocystis *sp. that are homologous to bacterial or archaeal genes, we identified 133 candidates for HGT (Table S3 in Additional file 2). In most cases, our phylogenetic analyses confirm the bacterial origin of these genes even if they were not sufficiently resolved to allow the precise identification of the donor, suggesting that these HGT events were ancient and/or that the corresponding genes are rapidly evolving in the genome of *Blastocystis *sp. Interestingly, in a few cases, even when the transferred gene is of bacterial origin, the *Blastocystis *sp. copy is closely related to homologues found in pathogenic and/or anaerobic eukaryotes, suggesting that HGT between eukaryotes has played a key role in these organisms too (Figure S6 in Additional file 1).

Some of the genes that originated from HGT possess functions that lead to a better understanding of how this lineage emerged. Three are homologous to the bacterial major facilitator transporter (MFS_1), the first two being nearly identical, and therefore resulting from a recent gene duplication event. MFS proteins form a large and diverse group of secondary transporters, which facilitate the transport across membranes of a variety of substrates, including ions, sugar phosphates, drugs, neurotransmitters, nucleosides, amino acids and peptides [43]. Two *Blastocystis *MFS genes have closely related homologues in some pathogenic eukaryotes like the Alveolata *Perkinsus marinus *or fungi such as *Gibberella zeae *and *Verticillium albo atrum*, suggesting an acquisition from bacteria followed by HGT between these eukaryotes (Figure S6f in Additional file 1). However, the phylogeny resolution is too low to precisely identify the bacterial donor of these genes. The presence of MSF proteins in *Blastocystis *sp. may confer the ability to absorb nutrients from the environment to this parasite, particularly in the intestinal lumen or when attacking host tissues. We have also found different HGT genes harboring alcohol deshydrogenase, short-chain dehydrogenase and oxidoreductase domains (Table S3 in Additional file 2) that may be involved in specific fermentations that remain to be characterized. Some of them are closely related to homologues found in anaerobic eukaryotes like *Trichomonas vaginalis *and *Entamoeba histolytica *(Figure S6b in Additional file 1) or in the bacteria *Legionella pneumophila *or *Parachlamydia acanthamoebae*, which infect or are associated with amoeba [44,45]. These enzymes may increase the range of *Blastocystis *sp. metabolic abilities to produce energy in anaerobic environments, as has been observed in *Giardia lamblia *and *E. histolytica *[46,47].

Several genes acquired by HGT may participate in the adhesion of the parasite to the host tissues. Indeed, 26 genes (Table S3 in Additional file 2) encode proteins containing the IPR008009 domain, which is often associated with immunoglobulin domains, a conserved core region of an approximately 90-residue repeat found in several hemagglutinins and other cell surface proteins. Among these 26 *Blastocystis *sp. proteins, some also contain the IPR015919 domain, which characterizes cadherins, a family of adhesion molecules that mediate Ca^2+^-dependent cell-cell adhesion. Homologous genes are also found in some beta-Proteobacteria or Acidobacteria, but the sequences are very divergent and our phylogenetic analysis did not, therefore, allow firm identification of the bacterial donor. Some hydrolase-encoding genes could also result from the transfer from bacteria to *Blastocystis *sp. One of them possesses an esterase-lipase (IPR013094) domain (Table S3 in Additional file 2) and may participate in the degradation of host tissue during infection. The closest homologues of this gene are found in the fungus *Botryotinia fuckeliana*, in Firmicutes and Actinobacteria (Figure S6d in Additional file 1).

Overall, these HGT genes may have allowed flexibility in genome expression, enabling the successful adaptation of *Blastocystis *sp. to digestive environments through genes encoding proteins that could be involved in osmotrophy (MFS), energy metabolism (dehydrogenases) and adhesion.

### Circular genome, predicted proteome and metabolic pathways of the MLOs

Although it lives in anaerobic or microaerophilic conditions, *Blastocystis *sp. harbors MLOs that present both mitochondrial and hydrogenosomal features [24]. We recently reported that *Blastocystis *sp. MLOs contain a circular genome, including genes encoding 10 of the 20 complex I subunits, but they lack all genes encoding cytochromes, cytochrome oxidases and ATP synthase subunits [24], unlike mitochondrial DNA from other sequenced stramenopiles, such as *Phytophthora *sp. [48]. The MLO genome of the *Blastocystis *subtype 7 is a circular molecule 29,270 bp in size. Two other MLO genomes were then sequenced from isolates belonging to other subtypes [49]: a subtype 1, represented by *Blastocystis *Nand II, with a 27,719 bp genome; and a subtype 4, represented by *Blastocystis *DMP/02-328, with a 28,382 bp genome. In addition to sequence conservation, these three genomes have many similarities. Their A+T content is around 80%, their gene density is higher than 95% and all three encompass 45 genes: 27 ORFs, 16 tRNAs and 2 rRNA genes. The ORFs consist of NADH subunits, ribosomal proteins and proteins with no similarity in the databases. The synteny between the three MLO genomes is highly conserved: gene order is strictly the same among the three genomes [24,49].

Through the analysis of a *Blastocystis *EST database, Stechmann *et al*. [23] have identified 110 potential proteins associated with mitochondrial pathways, such as the oxidative phosphorylation chain, tricarboxylic acid (TCA) cycle, Fe/S cluster assembly, and amino acid and fatty acid metabolisms. Nonetheless, approximately half of these proteins have an incomplete amino terminus due to EST data, making it difficult to confirm mitochondrial import by algorithms. To clarify the metabolic characteristics of these puzzling organelles, we used data from the whole genome sequence in order to establish the *in silico *proteome of *Blastocystis *MLOs. For this purpose, a computational approach based on two different prediction algorithms (MitoProt and MitoPred) for mitochondrial-import proteins was chosen (see Materials and methods for more details). This approach predicted 365 MLO proteins (Table S6 in Additional file 3) whereas Stechmann *et al*. [23] predicted only 110 proteins. Among these 365 proteins, 299 were predicted to have an amino-terminal extension involved in mitochondrial import, suggesting that an alternative system might exist for the 66 remaining proteins. Of the 299 proteins, 41 remain as 'hypothetical protein' with unknown function and 31 have no homologues in public databases, which raises the question of the existence of undiscovered metabolic processes within these intriguing organelles (Table S6 in Additional file 3). The other proteins are involved in classical mitochondrial core functions, such as oxidative phosphorylation, amino acid metabolism, fatty acid oxidation, iron-sulfur cluster assembly, and mitochondrial import system. Several proteins involved in the translocase of the outer mitochondrial membrane (TOM complex), the translocase of the inner membrane (TIM complex), and the presequence translocase-associated motor (PAM complex), which perform protein transport into the matrix, were identified. Interestingly, the two essential subunits of the mitochondrial processing peptidase heterodimer (MPP α/β), essential for the cleavage of the targeting peptide, were also found [50].

**Figure 2:**
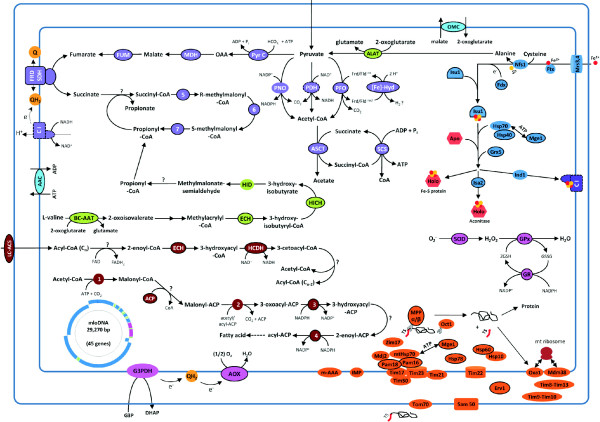
***In silico *reconstruction of metabolic pathways of *Blastocystis *sp. mitochondria-like organelles**. The proteins are predicted from the combined analysis of MitoProt and MitoPred algorithms. Proteins with a predicted amino-terminal extension are outlined by a solid black line, and protein complexes for which mitochondrial presequences for only some of the subunits have been predicted are outlined by a dashed black line. The pathways in purple represent: (1) the conversion of pyruvate into acetyl-CoA by the pyruvate dehydrogenase complex (PDH), pyruvate:ferredoxin oxidoreductase (PFO) or pyruvate:NADP oxidoreductase (PNO); (2) acetyl-CoA is then converted to acetate by acetate:succinate CoA transferase (ASCT) and may allow production of ATP (3). Pyruvate may follow routes that potentially use complexes I and II to produce succinate (and propionate) and certainly participate in maintaining the redox balance. The pathways in green and burgundy correspond to amino acid metabolism and fatty acid metabolism, respectively. Pathways for the assembly of iron-sulfur proteins are represented in blue, and proteins involved in mitochondrial import machinery in orange. Enzymes that may play a role in protection against oxidative stress are indicated in pink (superoxide dismutase (SOD), alternative oxidase (AOX), glutathione reductase (GR) and gluthathione peroxidase (GPx)); the role of glycerol-3-phosphate dehydrogenase (G3PDH) remains to be determined. Abbreviations: 1, acetyl-CoA carboxylase; 2, 3-oxoacyl-ACP synthase; 3, 3-oxoacyl-ACP reductase; 4, 2-enoyl-ACP reductase; 5, methylmalonyl-CoA mutase; 6, methylmalonyl-CoA epimerase; 7, propionyl-CoA carboxylase; AAC, ATP/ADP translocator; ACP, acyl carrier protein; ALAT, alanine aminotransferase; BC-AAT, branched-chain amino acid aminotransferase; C I, complex I; ECH, enoyl-CoA hydratase; [Fe]-Hyd, [Fe]-hydrogenase; FRD/SDH, fumarate reductase/succinate dehydrogenase activity of complex II; FUM, fumarase; HCDH, 3-hydroxyacyl-CoA dehydrogenase; HICH, 3-hydroxyisobutyryl-CoA hydrolase; HID, 3-hydroxyisobutyrate dehydrogenase; LC-ACS, long-chain acyl-CoA synthetase; MDH, malate dehydrogenase; OMC, oxoglutarate/malate carrier protein; Pyr C, pyruvate carboxylase; SCS, succinyl-CoA synthetase; SOD, superoxide dismutase.

Our analyses revealed that MLOs probably have three ways to make acetyl-CoA from pyruvate, supported by the presence of the pyruvate dehydrogenase complex, pyruvate:ferredoxin oxidoreductase and pyruvate:NADP^+ ^oxidoreductase (an amino-terminal pyruvate:ferredoxin oxidoreductase domain fused to a carboxy-terminal NADPH-cytochrome P450 reductase domain) (Figure 2). *Euglena gracilis *mitochondria include this feature, which provides adaptability to various oxygen levels [51], and this might be to a lesser extent the case for *Blastocystis *sp. We have also identified the 20 subunits of the *Blastocystis *sp. MLO complex I (ten are encoded by the MLO genome and ten by nuclear genes). The four nuclear-encoded subunits of the mitochondrial respiratory chain complex II were detected and this complex could function in two ways (via succinate dehydrogenase or fumarate reductase) [52]. We did not identify any genes encoding complexes III and IV subunits or ATP synthase. However, we have found components of the TCA cycle, which was shown to be involved with complex II (fumarate reductase) in fumarate respiration in parasitic helminths [52]. Interestingly, we identified a gene encoding a terminal oxidase, called alternative oxidase (AOX), which could be the terminal electron acceptor of complexes I and II (Figure 2), allowing adaptation to oxygen stress and maintaining the NADH/NAD balance, as has been suggested for *Cryptosporidium parvum *[53,54]. These data raise questions about the electron acceptor when complex II has succinate dehydrogenase or fumarate reductase activity, the quinone used in this process and the role of the proton gradient.

We also revealed proteins that can be grouped into essential mitochondrial pathways, like the Fe/S cluster assembly. More precisely, we have identified 11 enzymes (6 of which have predicted mitochondrial import signals), composing the iron-sulfur cluster system responsible for the assembly of mitochondrial Fe/S proteins [55], such as the cysteine desulfurase Nfs1, the scaffold protein Isu1, frataxin, and the P-loop NTPase Ind1, which is required for the assembly of complex I (Figure 2). We also highlighted some proteins involved in mitochondrial fatty acid synthesis type II [56], beta oxidation of fatty acids and amino acid metabolism (Table S6 in Additional file 3).

Taken together, our data confirm the mitochondrial nature of the *Blastocystis *sp. MLO. The oxygen-poor environment may have driven the selection of these unique organelles, which seemingly represent an intermediate situation between anaerobic mitochondria and hydrogenosomes, arguing for multiple situations arising during organelle evolution. It remains now to describe the metabolism occurring in these unusual organelles more precisely.

### Secretome and virulence factors

The persistence of *Blastocystis *sp. in the host may be due, to some extent, to its ability to override the response of the immune system and to adhere and survive within the intestinal tissue. Manipulation of the host might be facilitated by molecules released at the interface between the host and the parasite [57]. Accordingly, the study of the predicted secretome of *Blastocystis *sp. is of particular interest. With SIGNALP 3.0, 307 proteins were predicted to be secretory, of which 46 had no sequence similarity in the public nr databases. By sequence homology, 170 proteins that could play a role in host-parasite relationships were selected and submitted to PSORTII for extracellular location. Finally, 75 putative secreted proteins have been classified by putative functions, some of which may have a direct connection with pathogenicity (proteases, hexose digestion enzymes, lectins, glycosyltransferases and protease inhibitors; Table S4 in Additional file 2).

**Figure 3:**
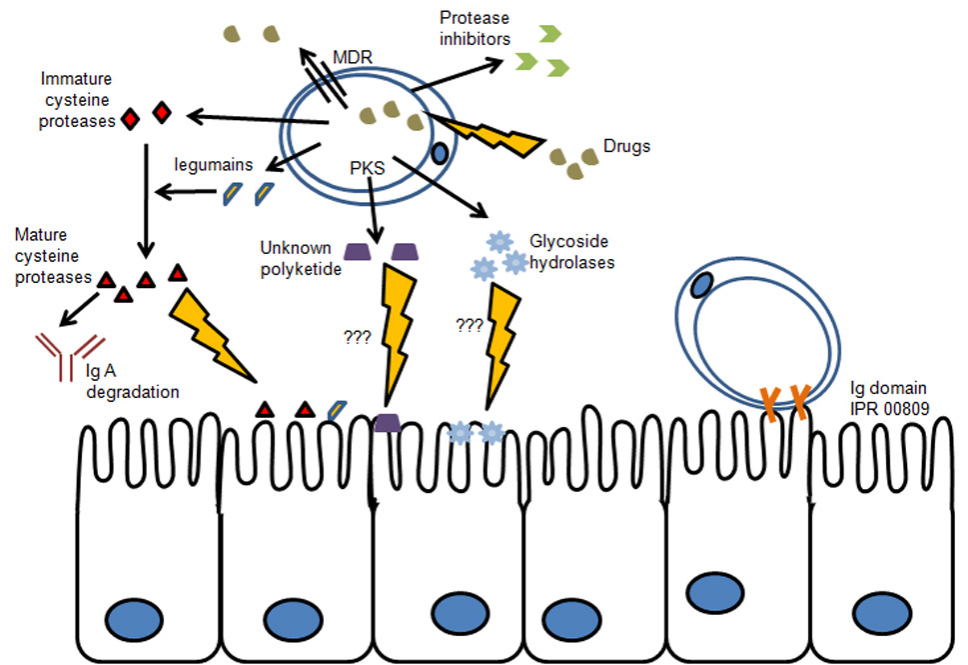
**Secretory proteins and virulence factors identified in the *Blastocystis *sp. subtype 7 potentially involved in host interaction**. *Blastocystis *sp. may release cysteine proteases, which could be processed by legumain. These proteases may attack intestinal epithelium together with other hydrolases, such as glysoside hydrolases. Protease inhibitors, some of which have been predicted to be secreted, could act on host proteases (digestive enzymes or proteases involved in the immune response). Some as yet uncharacterized secondary metabolites produced by polyketide synthase (PKS) identified in the genome could also participate in host intestinal symptoms. Adhesive candidate proteins (proteins with an immunoglobulin Ig domain) have been found. Finally, drug-resistant isolates of the parasite could be explained by the presence of multidrug resitance (MDR) proteins. Lightning bolts indicate potential toxic effects.

*Blastocystis *can secrete members of the immunophilin family, characterized by peptidyl-propyl *cis*-*trans *isomerase activity and disulfide isomerases (Figure 3; Table S4 in Additional file 2). These proteins have key roles in protein folding, but it has also been established that they can have moonlighting functions. In bacteria, they have evolved adhesive properties for the host [58] but they can also modulate host leukocyte function and induce cellular apoptosis [59]. A cyclophilin-like protein from the protozoan parasite *Toxoplasma gondii *is directly involved in host-parasite crosstalk, as it can modulate protective Th1 responses through its binding to the chemokine receptor CCR5 [60]. It is unclear what role these proteins play in *Blastocystis *sp., but this illustrates a range of functions for cell stress proteins in host-pathogen interactions.

Sugar-binding proteins have an important role through a conserved carbohydrate-recognition domain that could interact with host cell receptors. Such proteins have been characterized in other parasites [61] and it is interesting to note that some sugar-binding proteins are able to inhibit Th1- and Th2-mediated inflammation [62,63]. Moreover, some specific sugar-binding proteins are also able to suppress regulatory T cells [64]. The binding of these proteins is dependent on their specific sugar motifs, which can be added to N- or O-linked glycans by glycosyltransferases. One carbohydrate-binding protein and eight glycosyltransferases (Table S4 in Additional file 2) have been predicted to be secreted. All these enzymes could allow cross-linking of *Blastocystis *sp. sugar-binding proteins to host cell receptors.

The parasite likely uses hydrolases to attack host tissues. Fucosidase, hexosaminidase and polygalacturonase have been identified in the predicted secretome and may participate in this process by degrading host glycoproteins (Figure 3; Table S4 in Additional file 2). Proteases have been proposed to be involved in diverse processes, such as host cell invasion, excystation, metabolism, cytoadherence or other virulence functions. A correlation between a high level of protease activity and the virulence of the intestinal parasite *E. histolytica *was proven by McKerrow *et al*. [65]. Indeed, cysteine proteases degrade extracellular matrix proteins, cleave immunoglobulin A and G, and are thought to be responsible for the cytopathic effect of different pathogens against *in vitro *cultured cells [66]. Interestingly, *Blastocystis *sp. proteolytic enzymes are also able to degrade human secretory immunoglobulin A [67]. All the major classes of proteolytic enzymes were identified in the genome data, including serine, aspartic, and cysteine proteases and metalloproteases. Among the 66 proteases identified, 18 are predicted to be secreted by the parasite (Table S4 in Additional file 2). Within the protease family, cysteine protease-encoding genes are the most represented in *Blastocystis *sp. genome and 96% of the proteins encoded by these genes are predicted to be secreted. Among the cysteine proteases we have found five legumains and eight cathepsins; three cathepsins B contain the IPR015643 domain, which is only present in *Blastocystis *sp. compared to the other stramenopiles. The IPR015643 domain corresponds to the peptidase C1 cathepsin B domain and has a cysteine type peptidase activity, which was also found in pathogenic protozoa (*Leishmania *sp. and *Trypanosoma *sp.) [66]. Cysteine proteases are usually secreted in their inactive form and must be matured, having a prosegment that prevents hydrolysis during protease trafficking and storage. This maturation might result from the activity of the same protease or another, such as asparaginyl endopeptidase (also called legumain) [68]. This endopeptidase cleaves peptide bonds carboxy-terminal to asparagine residues, and may be involved in processing and activating both cathepsins L and B. Legumains have been predicted in the secretome of *Blastocystis *sp. (Table S4 in Additional file 2) and could be involved in protease processing (Figure 3). As an alternative role, secreted *Blastocystis *sp. legumains could also participate with other effectors in the alteration of the host intestine [69]. Indeed, it has been shown that legumain can degrade fibronectin, an extracellular matrix glycoprotein [70].

Genes coding for protease inhibitors are also present in the *Blastocystis *sp. genome, and some are predicted to be secreted. Release of protease inhibitors may weaken the host response as described in nematodes [71]. *Blastocystis *sp. encodes three protease inhibitors: cystatin, type1-proteinase inhibitor and endopeptidase inhibitor-like protein (Table S4 in Additional file 2). Type1-proteinase inhibitor is similar to chymotrypsin inhibitor, which is known to inactivate intestinal digestive enzymes (trypsin and chymotrypsin) as in *Ascaris suum *[72], thus protecting the parasite against non-specific digestive defenses. Cystatin, also called stefin, was described in *Fasciola gigantica *[73] and shown to inhibit mammalian cathepsin B, cathepsin L and other cysteine proteases, including parasite ones. In *Blastocystis *sp., secreted cystatin could participate in the regulation of parasitic cysteine protease activities. Cystatin can also potentially inhibit host proteases involved in MHC II antigen processing and presentation, including the key enzyme asparaginyl endopeptidase [74] and cathepsin S, the mammalian legumain [73].

Interestingly, a putative type I polyketide synthase (PKS) gene was also found in the *Blastocystis *sp. genome, potentially originating from HGT. PKS and non-ribosomal peptide synthetase (NRPS) synthesize metabolites like simple fatty acids, but also a myriad of chemical structures that possess important pharmacological activities and environmental impact, such as toxins, antibiotics or antimicrobials. Type I PKS was formerly known only from bacteria and fungi, but recently homologous genes were also discovered in some protists [75]. According to the Database for NRPS and PKS [76], the *Blastocystis *sp. *PKS *gene possesses the three essential domains, and three other domains: dehydratase, ketoacyl reductase, and enoyl reductase domains. The presence of these additional domains would permit this organism to synthesize both reduced polyketides and fatty acids. Domain comparison with other type I PKSs suggests that *Blastocystis *sp. PKS is similar to type I PKS from the ascomycete *Cochliobolus heterostrophus*, a maize pathogen that produces T toxin [77], a polyketide molecule that disturbs mitochondria by binding a protein of the inner mitochondrial membrane. Searching polyketide-related metabolites in the secretome of *Blastocystis *sp. would be of interest in order to identify molecules that could have effects on the host (Figure 3).

### Antioxidant system and multi drug resistance

Like other anaerobic organisms, *Blastocystis *sp. has to eliminate reactive oxygen species such as superoxide anions (O_2_^**.-**^), hydrogen peroxide (H_2_O_2_) and hydroxyl radicals (HO^.^) resulting from metabolism. In addition, this microorganism has to cope with the oxidative burst imposed by host immune cell effectors (release of O_2_^**.- **^subsequently processed to give additional reactive oxygen species). For these reasons, to protect against oxidative injury, *Blastocystis *species have developed an efficient battery of antioxidant enzymes (Table S5 in Additional file 2). The first lines of defense against oxygen damage are superoxide dismutases (SODs), a family of metalloproteins catalyzing the dismutation of O_2_^**.- **^to form H_2_O_2 _and oxygen. Genome annotation revealed the presence of two genes encoding SODs (SOD1 and SOD2) that exhibit sequence characteristics of dimeric iron-containing SODs [78] and likely protect the cytosol and MLOs, respectively, against O_2_^**.-**^. Catalase and ascorbate peroxidase are subsequently able to remove H_2_O_2 _generated by SODs as well as by NADPH-dependent oxidase. However, genes encoding catalase and ascorbate peroxidase have not been identified in *Blastocystis *sp. nor in many unicellular parasites, including trypanosomatids and *Plasmodium falciparum*. Additional enzymes, glutathione peroxidase (Gpx) and thioredoxin-dependent peroxidase (commonly known as peroxyredoxin (Prx)) are able to reduce H_2_O_2 _to water as well as other substrates, such as hydroperoxides and peroxinitrite. In most eukaryotes, both enzymes obtain their reducing equivalents from two redox systems, the glutathione (GSH) and the thioredoxin (Trx) systems, respectively. Like *P. falciparum *[79], *Blastocystis *sp. cells possess a complete GSH synthesis pathway: the genes encoding γ-glutamylcysteine synthetase, glutathione synthetase (eu-GS group) and a functional GSH/Gpx (nonselenium Gpx belonging to the PHGpx group)/glutathione reductase system have been identified and both Gpx and glutathione reductase are probably located in the MLO. This nearly ubiquitous redox cycle is replaced by the trypanothione system in trypanosomatids [80]. *Blastocystis *sp. also contains genes encoding the proteins of the Trx/thioredoxin reductase (TrxR)/Prx system. Indeed, two genes encode small proteins homologous to Trx: one cytosolic and another most likely located in the MLO (Table S5 in Additional file 2). Trx is itself reduced by TrxR and three genes encoding cytosolic TrxR have been identified in *Blastocystis *sp. These proteins clearly belong to the high molecular weight (designated H-TrxR) group of enzymes and are similar to metazoan enzymes, including those of *Homo sapiens *and *Drosophila melanogaster*, and to those of the apicomplexan protozoa *Plasmodium*, *Toxoplasma*, and *Cryptosporidium *[81]. Interestingly, in contrast to apicomplexan H-TrxRs, two of the H-TrxR enzymes of *Blastocystis *are predicted to possess a redox active center in the carboxy-terminal domain composed of a selenocysteine (a rare amino acid encoded by the opal codon TGA, which is not recognized as a stop codon) at the penultimate position and its neighboring cysteine residue as in metazoan enzymes (selenoprotein type H-TrxR). This strongly suggests the presence of the Se-Cys insertion machinery (SECYS elements) in *Blastocystis *sp. Genes encoding another type of TrxR with low molecular weight (designated L-TrxR) have been identified in parasitic protozoa such as *Trichomonas*, *Entamoeba*, and *Giardia *but not in the genome of *Blastocystis *sp. These data reinforce the assumption of the exclusive occurrence of either L-TrxR or H-Trxr in genomes and of some disadvantages of possessing both types of TrxR [81]. In *Blastocystis *sp., at least 11 highly similar gene copies encoding predicted cytosolic Prxs have been found that clearly belong to the typical 2-Cys class of Prx. Whether sequence polymorphism of these enzymes is potentially correlated with diversified expression or even function remains to be explored. Another gene encoding a typical 2-Cys Prx, likely located in the MLO, has been identified in this parasite. Interestingly, like the homologous sequence of another stramenopile, *P. infestans*, this latter protein is fused to Trx with a WCGKC motif. As described above, *Blastocystis *sp. possesses a whole array of antioxidant enzymes protecting both the cytosol and MLO. As shown in Table S5 in Additional file 2, these enzymes have distinct phylogenetic origins and most of them probably originate from prokaryote HGT. These antioxidant proteins attract attention in unicellular parasites as they have important functions in host-parasite interactions and constitute new drug targets for the design of inhibitors. Indeed, genetic approaches have undoubtedly shown that some anti-oxidant enzymes are essential for the survival of different parasitic species [82-86].

Some genes coding for multi-drug resistance pump proteins have also been discovered in the *Blastocystis *sp. genome. There are two classes of multi-drug resistance genes: the first class corresponds to proteins that are energized by ATP hydrolysis; the second class includes proteins that mediate the drug efflux reaction with a proton or sodium ion gradient. Among the first class, 24 ABC transporter genes were found. In eukaryotes the main physiological function of ABC transporters is the export of endogenous metabolites and cytotoxic compounds [87] and eight families of ABC transporters (ABC A to H) have been identified. The *Blastocystis *sp. ABC transporters are included in four of these eight families (five in family A, six in family B, six in family C, three in family F, and four not in any class). The A family is involved in lipid trafficking, and the F family in DNA repair and gene regulation. The other two families are more interesting [87], since in protozoan parasites (*Leishmania *spp., *Trypanosoma *spp., *Plasmodium *spp.) transporters belonging to the B and C families confer resistance to drugs. Metronidazole-resistant strains of *Blastocystis *sp. could have arisen through the action of these multi-drug resistance proteins (Figure 3).

## Conclusions

We have provided the first genome sequence of a *Blastocystis *sp. subtype, which could serve in comparative genomics studies with other subtypes to provide clues to clarify how these protozoans develop pathogenicity in some humans. Analysis of this genome has revealed original traits of this lineage compared to other stramenopiles (free living and plant pathogens). Aerobic respiration has been lost, *Blastocystis *sp. instead having the MLO, an anaerobic organelle, which should advance our understanding of organelle evolution as the *Blastocystis *sp. MLO seems to be unique among organelles (Figure 2) but remains to be biochemically characterized. Some genes may have been gained through HGT, which may participate in essential functions for an intestinal parasite (adhesion, energy production). These genes probably have facilitated adaptation to intestinal environments. The *Blastocystis *sp. secretome has been predicted and this has permitted the identification of candidate proteins that could degrade host tissues in order to provide nutrients. Putative secretory proteins that can interfere with non-specific and specific host defense systems have also been found, enabling *Blastocystis *sp. to survive within this hostile environment (Figure 3). These putative secretory proteins are of particular interest as they may interact directly with host tissue and could help in understanding the host-parasite interactions and could also be used as markers to distinguish between non-pathogenic and pathogenic isolates. If their functions are essential, they could also be used to develop future vaccine formulations. The antioxidant proteins offer interesting therapeutic targets as they might be important for the parasite in fighting oxidative bursts. In summary, the deciphering of the *Blastocystis *sp. genome will contribute to the study of interactions between this parasite and its host at a post-genomic scale and pave the way for deciphering the host-parasite interactome. Finally, the '*Blastocystis *sp. story' is reminiscent of the amoeba pathogenicity story where two morphologically indistinguishable species have different pathogenic potential [88], and this genome will help in the development of typing tools for the characterization of pathogenic isolates.

## Materials and methods

### Genome sequencing

The *Blastocystis *sp. genome was sequenced using a whole genome shotgun strategy. All data were generated by paired-end sequencing of cloned inserts using Sanger technology on ABI3730xl sequencers. Table S1 in Additional file 2 gives the number of reads obtained per library. All reads were assembled with Arachne [89]. We obtained 157 contigs that were linked into 54 supercontigs. The contig N50 was 297 kb, and the supercontig N50 was 901 kb (Table S2 in Additional file 2).

### Genome annotation

#### Construction of the training set

A set of 300 gene models from a preliminary annotation run was selected randomly, among those that were validated by *Blastocystis *sp. cDNAs (that is, with every intron confirmed by at least one cDNA and no exon overlapping a cDNA intron) to create a clean *Blastocystis *sp. training set. This training set was used to train gene prediction algorithms and optimize their parameters.

#### Repeat masking

Most of the genome comparisons were performed with repeat masked sequences. For this purpose, we searched and masked sequentially several kinds of repeats: known repeats and transposons available in Repbase with the Repeat Masker program [90], tandem repeats with the TRF program [91], *ab initio *repeat detection with RepeatScout [92], rDNA by BLATing [93] 189 rDNAs sequences (downloaded from GenBank), and telomeric repeats by searching 'CCCTAA' patterns in the scaffolds with the BLAST2 algorithm.

#### GeneWise

The UniProt [94] database was used to detect conserved genes between *Blastocystis *sp. and other species. As GeneWise [95] is time greedy, the UniProt database was first aligned with the *Blastocystis *sp. genome assembly using BLAT [93]. Subsequently, we extracted the genomic regions where no protein hit had been found by BLAT and realigned Uniprot proteins with more permissive parameters. Each significant match was then refined using GeneWise in order to identify exon/intron boundaries.

#### GeneID and SNAP

GeneID [96] and SNAP [97]*ab initio *gene prediction software were trained on 300 genes from the training set.

#### Blastocystis sp. cDNAs

Full-length-enriched cDNA libraries were constructed from *Blastocystis *sp. vacuolar forms using a SV total RNA isolation system (Promega France, Charbonnières, France) for RNA extraction. RNA quality and quantity were estimated using the Agilent bioanalyser with the RNA 6000 Nano LabChip^® ^Kit. The clones were sequenced on the 5' end, producing 34,470 useful reads. We were able to align 33,685 cDNA sequences to the *Blastocystis *sp. genome assembly with the following pipeline: after masking of polyA tails, the sequences were aligned with BLAT on the assembly and all matches with scores within 99% of the best score were extended by 5 kb on each end, and realigned with the cDNA clones using the EST2genome software [98].

#### Stramenopile ESTs

A collection of 410,069 public mRNAs from the stramenopile clade (276,208 downloaded from the National Center for Biotechnology Information plus 43,932 and 80,929 ESTs downloaded from the Joint Genome Institute for diatoms and *Ectocarpus*, respectively) were first aligned with the *Blastocystis *sp. genome assembly using BLAT [93]. To refine BLAT alignment, we used EST2genome [98]. Each significant match was chosen for an alignment with EST2genome. BLAT alignments were made using default parameters between translated genomic and translated ESTs.

#### Integration of resources using GAZE

All the resources described here were used to automatically build *Blastocystis *sp. gene models using GAZE [99]. Individual predictions from each of the programs (that is, GeneID, SNAP, GeneWise, EST2genome) were broken down into segments (coding, intron, intergenic) and signals (start codon, stop codon, splice acceptor, splice donor, transcript start, transcript stop).

Exons predicted by *ab initio *software (that is, GeneWise and EST2genome) were used as coding segments. Introns predicted by GeneWise and EST2genome were used as intron segments. Intergenic segments were created from the span of each mRNA using a negative score (coercing GAZE not to split genes). Predicted repeats were used as intron and intergenic segments to avoid prediction of genes coding proteins in such regions.

The whole genome was scanned to find signals (splice sites and start and stop codons). Additionally, transcript stop signals were extracted from the ends of mRNAs (polyA tail positions).

Each segment extracted from software output that predicts exon boundaries (like GeneWise, Exonerate or *ab initio *predictors) was used by GAZE only if GAZE chose the same boundaries. Each segment or signal from a given program was given a value reflecting our confidence in the data, and these values were used as scores for the arcs of the GAZE automaton. All signals were given a fixed score, but segment scores were context sensitive: coding segment scores were linked to the percentage identity of the alignment; intronic segment scores were linked to the percentage identity of the flanking exons. A weight was assigned to each resource to further reflect its reliability and accuracy in predicting gene models. This weight acts as a multiplier for the score of each information source, before processing by GAZE. When applied to the entire assembled sequence, GAZE predicted 4,798 gene models. Since the resource of expressed sequences in stramenopiles is limited, and some gene-free 'holes' appeared in gene-dense regions, we suspected that some genes had been missed by the annotation pipeline because of a lack of support.

#### Additional gene models

With the assumption that not all genes in *Blastocystis *sp. have EST support, we developed the following strategy to recuperate additional gene models. *Ab initio *(SNAP and GeneID) predictions that did not overlap GAZE gene models were selected and aligned to UniProt sequences. Predictions that had significant hits (coverage ≥90%; e-value ≤10^-5^) were tagged as potential coding genes and randomly chosen genes were successfully verified by RT-PCR using the Access RT-PCR system (Promega France, Charbonnières, France). The final proteome composed of 6,020 gene models was obtained by adding 1,222 supplementary models to the 4,798 genes from the first GAZE output.

### Identification of orthologous genes

We identified orthologous genes with three species: *Cyanidioschyzon merolae *[100], *P. sojae *[49] and *T. pseudonana *[101]. Each pair of predicted genes was aligned with the Smith-Waterman algorithm, and alignments with a score higher than 300 (BLOSUM62, gapo = 10, gape = 1) were retained. Orthologs were defined as BRHs, that is, two genes, A from genome GA and B from genome GB, were considered orthologs if B is the best match for gene A in GB and A is the best match for B in GA.

### Identification of paralogous genes and duplicated blocks

An all-against-all comparison of *Blastocystis *sp. proteins was performed using the Smith-Waterman algorithm implemented in the Biofacet package [102]. BRHs were identified as follows: two genes, A and B, are the BRH if B is the best match for gene A and A for gene B. The distribution of percentage identities among the pairs of BRHs is displayed in Figure S7 in Additional file 1. The distribution is widespread except for the abundant class of genes sharing ≥90% of identity, which represents 48% of all pairs of paralogs. We investigated this apparently recent gene duplication by selecting all pairs of genes sharing ≥90% identity over ≥50% of the length of the shortest protein (not only BRHs), which gave 1,917 gene pairs corresponding to 1,141 genes scattered in 404 gene families (19% of *Blastocystis *sp. genes). The number of counterparts per gene is displayed in Figure S2 in Additional file 1. Additionally, blocks of paralogous genes, or so-called duplicated blocks, were identified by clustering the 1,917 gene pairs. The clustering was performed by single linkage clustering using the Euclidian distance between genes, and independently of gene orientation. Those distances were calculated with the gene index on each scaffold rather than the genomic position, including only the genes with paralogs. The minimal distance between two paralogous genes was set to 5 and the minimal number of genes in a cluster was set to 4 (two pairs of paralogous genes; Figure S8 in Additional file 1).

### Identification of candidate horizontal gene transfers

*Blastocystis *sp. proteins were blasted [103] (blastx) against the protein nr database with the parameters '-f 100 -X 100 -e 0.00001 -E 2 -W 5', and the best hits were retained using the following criteria: for BLAST scores greater than 200, all hits with a score greater than 90% of the best score were retained; and for BLAST scores lower or equal to 200, all hits with a score greater than 80% of the best score were retained. Then, the proteins with all their best hits in bacteria or archaea were retained as candidates that had potentially arisen from HGT. Other criteria for the blastx comparison were tested (such as W = 3) but we observed no significant difference in the results after the subsequent filters. Candidates with some of their best hits in stramenopiles in addition to bacteria were also retained since some HGTs may be shared between stramenopiles, and genes for which orthologs were identified in non-stramenopile species were discarded. The evolutionary origin of the candidate genes was then investigated using phylogenetic approaches (Figure S6 in Additional file 1). For each gene, homologues were retrieved from the protein nr database using Blastp (default parameters, except for the max-target-sequences threshold, which was fixed at 500). The sequences were aligned using Muscle 3.6 [104] (default parameters). The resulting alignments were visually inspected and manually refined using the MUST software [105]. Ambiguously aligned regions were removed prior to phylogenetic analysis.

Maximum likelihood phylogenetic tree reconstructions were carried out on the remaining positions using PhyML [106] with the Le and Gascuel (LG) model [106] with a gamma correction (four discrete classes, an estimated alpha parameter) to take into account evolutionary rate variation among sites. Tree robustness was estimated by a non-parametric bootstrap approach using PhyML and the same parameters with 100 replicates of the original dataset. Bayesian phylogenetic trees were also reconstructed using MrBayes version 3.1.2 [107]. We used a mixed model of amino acid substitution and a gamma distribution (four discrete categories plus a proportion of invariant sites) to take into account site rate variation. MrBayes was run with four chains for 1 million generations and trees were sampled every 100 generations. To construct the consensus tree, the first 1,500 trees were discarded as 'burn-in'. The candidates with clear eukaryotic origin were then discarded. This process provided 133 candidate genes (Table S3 in Additional file 2). These candidates contain a high proportion of monoexonic genes (39%) compared to the average number of monoexonic genes in *Blastocystis *sp. (approximately 15%).

### Protein domain analysis

InterProScan [108] was run against all *C. merolae*, *P. sojae*, *T. pseudonana *and *Blastocystis *sp. proteins. Matches that fulfilled the following criteria were retained: match tagged as 'true positive' by InterProScan (status = T); match with an e-value ≤10^-1^. A total of 2,305 InterPro domains (with IPR number) were found in *Blastocystis *sp., which corresponds to 4,096 proteins.

### Functional annotation

#### Enzyme annotation

Enzyme detection in predicted *Blastocystis *sp. proteins was performed with PRIAM [109], using the PRIAM July 2006 Enzyme release. A total of 428 different EC numbers, corresponding to enzyme domains, are associated with 1,140 *Blastocystis *sp. proteins. Therefore, about 19% of *Blastocystis *sp. proteins contain at least one enzymatic domain.

#### Association of metabolic pathways with enzymes and *Blastocystis *sp

Potential metabolic pathways were deduced from EC numbers using the KEGG pathway database [110]. Links between EC numbers and metabolic pathways were obtained from the KEGG website. Using this file and the PRIAM results, 906 (of the 1,140) *Blastocystis *sp. proteins were assigned to 201 pathways.

#### Identification of putative proteins imported within the MLOs

The whole proteome was scanned by two algorithms aimed at predicting proteins imported to mitochodria; MitoProt [111], which predicts mitochondrial-targeting sequences, and MitoPred [112], which predicts nuclear-encoded mitochondrial proteins based on Pfam domains (animal/yeast database). After manual processing and using a script, only protein sequences with a score above 0.5 and 85% for MitoProt and MitoPred, respectively, were selected. This output file was then used in a KEGG Automatic Annotation Server (KAAS) with the bi-directional best hit method [113] in order to automatically generate KEGG pathways. Because protein domain annotations did not always provide sufficient information (PRIAM July 2006 Enzyme release), a BLAST comparison against the non-redundant database was conducted.

### Secretome prediction using SignalP 3.0 and pSORTII

Prediction of secreted proteins is based on the analysis of amino-terminal secretory signal sequences (SignalP 3.0) followed by the selection of proteins predicted as extracellular by pSORTII. Each of the proteins was individually submitted to SignalP 3.0 for analysis with the following parameters: organism set to eukaryotes, output short format and protein sequence truncation after the first 50 amino acids. Results of SignalP 3.0 were exported to a temporary file, and identification of signal peptides was accomplished by parsing the results of the hidden Markov model analysis conducted by SignalP 3.0. Proteins with secretory signals were retained and analyzed on the basis of possible function in host-parasite interactions. These last ones were also analyzed using PSORT II [114], and those having a best hit as 'extracellular' were selected. The SignalP threshold value for secretory signal peptide prediction was set at 0.5 as determined for previous analyses [115] and the best hit was chosen for the PSORTII analysis. The predicted secretory proteins were then annotated as functional protein families.

## Abbreviations

bp: base pair; BRH: best reciprocal hit; EST: expressed sequence tag; Gpx: glutathione peroxidase; GSH: glutathione; HGT: horizontal gene transfer; KEGG: Kyoto Encyclopedia of Genes and Genomes; MFS: major facilitator transporter; MLO: mitochondria-like organelle; NRPS: non-ribosomal peptide synthase; ORF: open reading frame; PKS: polyketide synthase; Prx: peroxyredoxin; SOD: superoxide dismutase; TCA: tricarboxylic acid; Trx: thioredoxin; TrxR: thioredoxin reductase; WGD: whole genome duplication.

## Competing interests

The authors declare that they have no competing interests.

## Authors' contributions

FDen, PW, CPV and HEA conceived and designed the experiments. MR, IW, JP, GCN, BS, BN, CDS, AC and HEA performed the experiments. MR, IW, MD, CT, BN, EV, CBA, FDen, VA, FA, JMA, OJ, KSWT, FDel, PW and HEA analyzed the data. FDen, MR, IW, EV, CBA, FDel, CPV and HEA wrote the paper. All authors read and approved the final manuscript.

## Supplementary Material

Additional file 1 **Genome organization of *Blastocystis *sp. (introns, numbers of counterparts per gene, genome structure, and so on) and phylogenetic trees illustrating horizontal gene transfer events from prokaryotic donors to *Blastocystis *sp. and candidate genes for endosymbiotic gene transfers of chloroplastic origin**. Click here for file

Additional file 2 **Sequencing overview and assembly metric data, and the identification of horizontal gene transfer, secretory protein and antioxidant protein candidates**. Click here for file

Additional file 3 **Proteins putatively imported in the mitochondria-like organelle**. Click here for file
