# Supplementary material for: Genome sequence of the stramenopile Blastocystis, a human anaerobic parasite
Source: Genome Biol. 2011 Mar 25;12(3):R29. doi: 10.1186/gb-2011-12-3-r29 (PMC3129679; doi:10.1186/gb-2011-12-3-r29)
Supplement: Additional file 1 — Genome organization of Blastocystis sp. (introns, numbers of counterparts per gene, genome structure, and so on) and phylogenetic trees illustrating horizontal gene transfer events from prokaryotic donors to Blastocystis sp. and candidate genes for endosymbiotic gene transfers of chloroplastic origin. [file gb-2011-12-3-r29-S1.DOC]

**Additional genomic and phylogenetic data**

**Supplementary figures**

**A.**


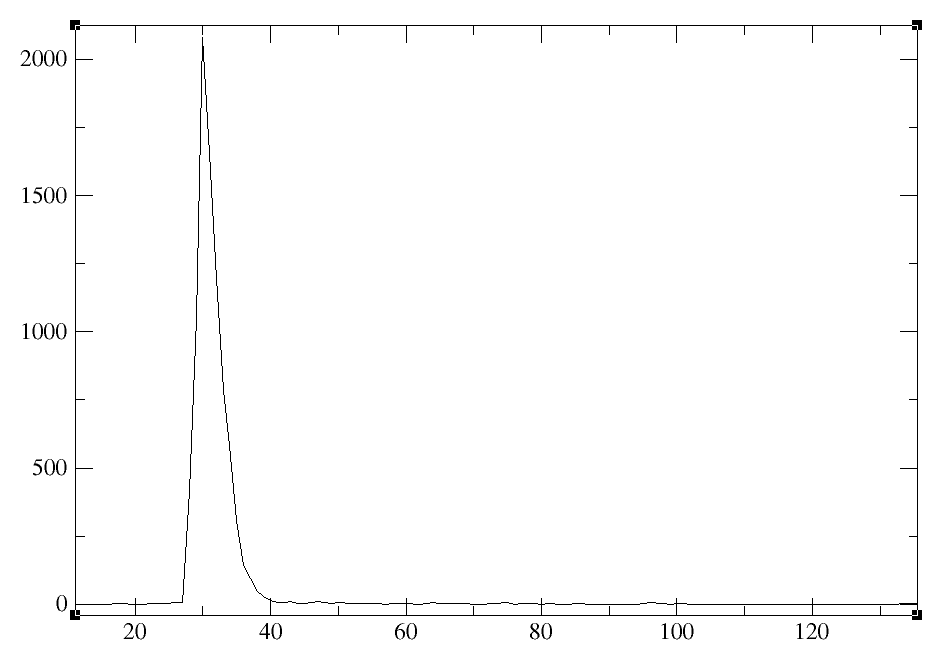


**B.**

**
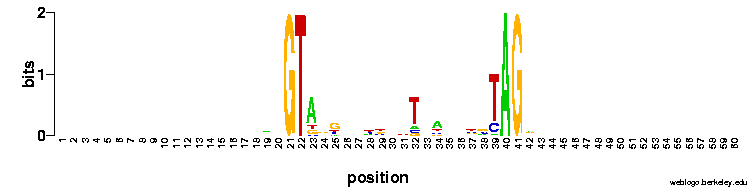
**

**Figure S1. Characteristics of *Blastocystis* *sp.* introns.**

A. Intron length distribution for 8569 introns validated by cDNAs.

B. Intron logos, obtained with Weblogo.

**
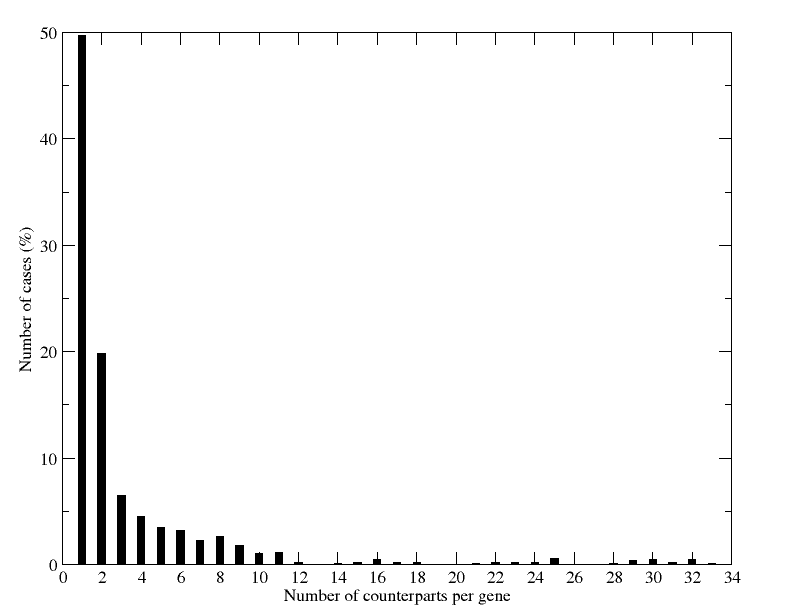
**

**Figure S2. Number of counterparts per gene**

**
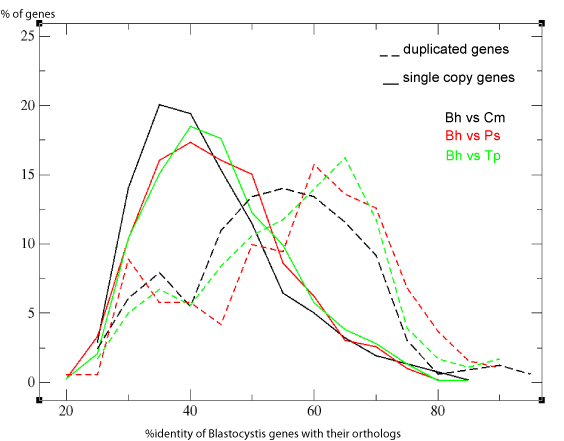
**

**Figure S3. Identity percentages of *Blastocystis* *sp.* single copy genes and duplicated genes with their orthologs in 3 species (*Cyanidioschyzon merolae*, *Phytophthora sojae* and *Thalassiosira pseudonana*).**

**
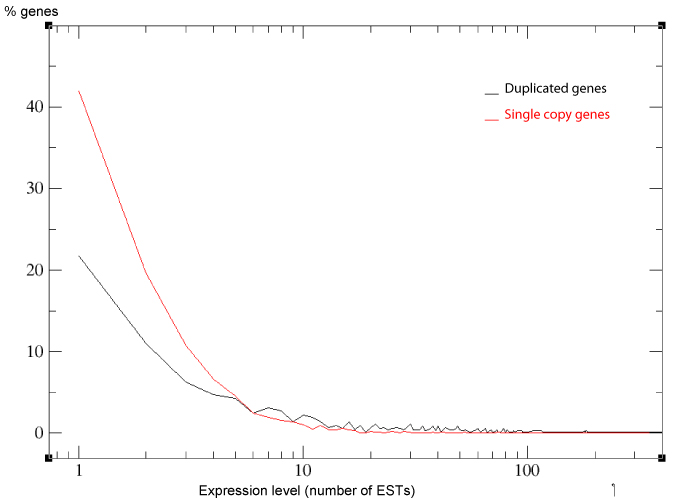
**

**Figure S4. Expression level of single copy and duplicated genes**

**Figure S5. Maximum Likelihood phylogenetic trees illustrating candidate gene for endosymbiotic gene transfers of chloroplastic origin.** (A) phosphoglycerate kinase, (B) 6-phosphogluconate dehydrogenase. The scale bars represent the inferred average number of substitutions per site. Numbers at nodes represent bootstrap values (100 replicates of the original datasets). For clarity only values greater that 50% are shown.

**Figure S6 : Maximum likelihood (left tree) and Bayesian (right tree) phylogenetic trees illustrating HGT events from prokaryotic donors to *Blastocystis*:** (a) iron-sulfur cluster assembly SUFB (GSBLHT00004911001, 50 sequences, 226 amino acid positions), (b) metal ion binding alcohol dehydrogenases  (GSBLHT00004776001, GSBLHT00004433001, GSBLHT00002924001, 76 sequences, 275 amino acid positions), (c) hydrolase (GSBLHT00002439001, 50 sequences, 145 amino acid positions), (d) hydrolase (GSBLHT00001822001, 48 sequences, 131 amino acid positions), (e) hydrolase (GSBLHT00000797001, 67 sequences, 130 amino acid positions), (f) MSF (GSBLHT00000637001, 67 sequences, 222 amino acid positions). Archaeal sequences are indicated in red, bacterial sequences in black, viral sequences in brown, eukaryotic sequences in dark blue and *Blastocystis* sequences in light blue. The trees were reconstructed using PhyML with the LG model with estimated amino frequencies, a gamma-correction (4 discrete categories and an estimated alpha-parameter). The scale bar represents the average number of substitutions per site. Numbers at nodes correspond to bootstrap values (for clarity only those >50% are shown).


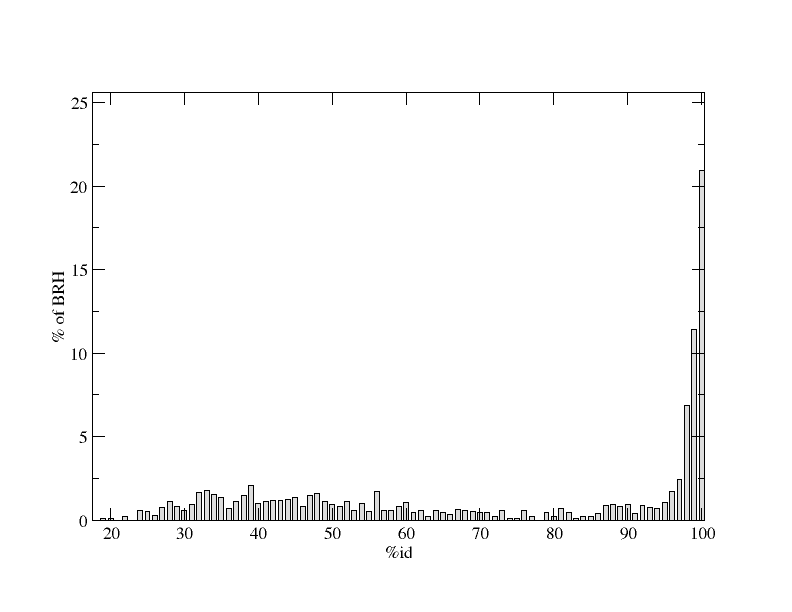


**Figure S7. Distribution of %ids among the pairs of BRHs**

**
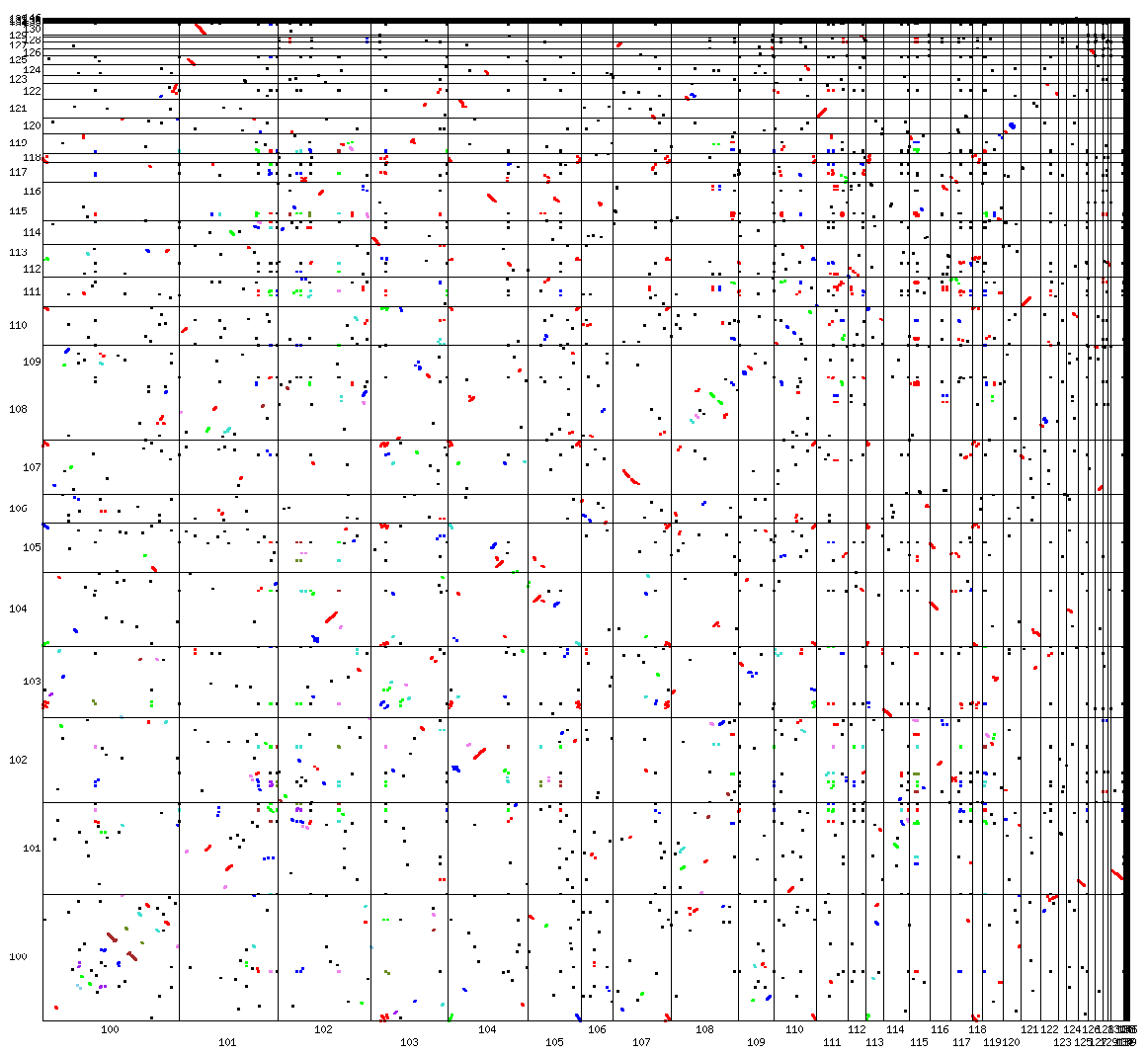
**

**Figure S8. Identification of duplicated blocks.** The pairs of paralogous genes were plotted (the positions used are the gene index -among paralogous genes- rather than genomic positions). Then, we clustered together the pairs of paralogous genes that were distant by less than 5 genes on both counterparts and only retained clusters that were composed of at least 3 pairs of paralogous genes. Genes belonging to clusters are colored; genes in black do not belong to clusters.
