# Supplementary material for: Genome sequence of the stramenopile Blastocystis, a human anaerobic parasite
Source: Genome Biol. 2011 Mar 25;12(3):R29. doi: 10.1186/gb-2011-12-3-r29 (PMC3129679; doi:10.1186/gb-2011-12-3-r29)
Supplement: Additional file 2 — Sequencing overview and assembly metric data, and the identification of horizontal gene transfer, secretory protein and antioxidant protein candidates. [file gb-2011-12-3-r29-S2.PDF]

Sequencing overview, assembly metrics and identification of horizontal gene transfer, secretory protein and antioxidant protein candidates

Supplementary tables

Table S1. Sequencing overview.

Library name	Type	Insert sizes	Number of reads	Coverage
AAA	plasmid, high-copy	3 kb	164,381	6.7 x
AAC	BAC	30 kb	7,337	0.3 x
ABB	plasmid, low-copy	10 kb	61,589	2.5 x
ACA	plasmid, low-copy	10 kb	72,250	2.9 x
total			305,557	12.4 x

Table S2. Assembly metrics.

	Contigs	Scaffolds
Number	157	54
Cumulative size	18,743,698	18,808,479
Average size	355,610	993,492
N50 size	296,810	900,598
Largest	900,598	1,966,360

Table S3. *Blastocystis* sp. candidate horizontal gene transfers.

Gene id	Number of exons	Interpro domains	GO	
GSBLHT00001948001	1	IPR002903	methyltransferase activity	Bacteria (6 Firmicutes + 3 Bacteroidetes)
GSBLHT00003862001	1	IPR006056,IPR006175,IPR013813	-	Bacteria (1 Archaea)
GSBLHT00001595001	5	IPR000589,IPR005290,IPR009068	translation	Bacteria (2 Firmicutes + 1 Eukaryotes/Heterolobosea)
**GSBLHT00004776001**	**4**	**IPR001670 : ALCOHOL DEHYDROGENASE, IRON TYPE : alcohol dehydrogenase ; iron type**	**metal ion binding**	**Bacteria (1 Eukaryotes/Parabasalia)**
**GSBLHT00002924001**	**4**	**IPR001670 : ALCOHOL DEHYDROGENASE, IRON TYPE : alcohol dehydrogenase ; iron type**	**metal ion binding**	**Bacteria/Eukaryotes (Eukaryotes/Amoebozoa,Parabasalia)**
GSBLHT00000298001	1	IPR001732,IPR008927,IPR014026,IPR014027,IPR014028,IPR016040,IPR017476	NAD binding	Bacteria (Fusobacteria, Firmicutes, Gammaproteobacteria)
GSBLHT00001520001	1	IPR001732,IPR008927,IPR014026,IPR014027,IPR014028,IPR016040,IPR017476	NAD binding	Bacteria
**GSBLHT00004433001**	**1**	**IPR001670 : ALCOHOL DEHYDROGENASE, IRON TYPE**	**metal ion binding**	**Bacteria**
GSBLHT00001492001	1	IPR004550,IPR006034	asparagine metabolic process	Bacteria
**GSBLHT00001543001**	**3**	**IPR002085 ALCOHOL DEHYDROGENASE**	**oxidoreductase activity**	**Bacteria (Parachlamydia)**

		**SUPERFAMILY, ZINC-CONTAINING, IPR011032 , IPR013149 ALCOHOL DEHYDROGENASE, C-TERMINAL,IPR013154, IPR016040**		
GSBLHT00003844001	1	IPR006314	-	Betaproteobacteria/Gammaproteobacteria
GSBLHT00003538001	2	IPR000600,IPR007260,IPR011060,IPR013785	N-acylglucosamine-6-phosphate 2-epimerase activity	Bacteria (4 Fusobacteria + 9 Firmicutes + 1 Actinobacteria + 2 Bacteroidetes)
GSBLHT00006031001	6	IPR001395	oxidoreductase activity	Bacteria (2 Eukaryotes/Metazoa)
GSBLHT00000076001	5	IPR002876,IPR017856	-	Bacterial (Bacteroidetes)
GSBLHT00006331001	5	IPR002523	metal ion transmembrane transporter activity	Bacteria
**GSBLHT00004535001**	**9**	**IPR001670 : ALCOHOL DEHYDROGENASE, IRON TYPE**	**metal ion binding**	**Bacteria**
GSBLHT00003833001	5	IPR001279	hydrolase activity	Bacteria (1 Eukaryotes/Parabasalida)
GSBLHT00002220001	3	IPR003702	acetyl-CoA metabolic process	Deltaproteobacteria (Deltaproteobacteria and Eukaryotes/Trichomonas vaginalis)
GSBLHT00000510001	3	IPR003702	acetyl-CoA metabolic process	Deltaproteobacteria (Deltaproteobacteria and Eukaryotes/Trichomonas vaginalis)
GSBLHT00000797001	3	IPR001279	hydrolase activity	Bacteria (Various bacteria)
GSBLHT00000853001	2	IPR005130,IPR005131	-	Bacteria (various bacteria + 4 Diplomonadida)
GSBLHT00002333001	3	IPR005130,IPR005131	-	Bacteria (various bacteria + 4 Diplomonadida)
GSBLHT00004011001	5	IPR002917,IPR009019,IPR015946	Intracellular	Eukaryotes/Alphaproteobacteria
GSBLHT00001846001	4	IPR005130,IPR005131	-	Bacteria/Eukaryotes (Diplomonadida)

GSBLHT00006705001	2	IPR001227,IPR016035	transferase activity	Firmicutes
GSBLHT00004472001	3	IPR001789,IPR003594,IPR005467,IPR011006	peptidyl-histidine phosphorylation	Bacteria (1 Gammaproteobacteria + 1 Deltaproteobacteria + 1 Chlorofexi)
GSBLHT00003380001	5	IPR002220,IPR013785	lyase activity	Bacteria (1 Actinobacteria)
GSBLHT00000282001	5	IPR003140	-	Alphaproteobacteria/Eukaryotes
GSBLHT00001568001	1	-	-	Bacteria
GSBLHT00001190001	1	IPR005502	-	Bacteria likely Actinobacteria
GSBLHT00004537001	6	IPR008183,IPR011013,IPR014718,IPR015443	carbohydrate binding	Gammaproteobacteria
**GSBLHT00004388001**	**1**	**IPR008009,IPR015919**	**Membrane**	**Homologues are too divergent, but likely bacterial**
GSBLHT00000807001	2	-	-	Cyanobacteria/Fungi
GSBLHT00000159001	1	IPR009784	-	Bacteria (Firmicutes)
GSBLHT00006479001	1	IPR005900	6-phosphogluconolactonase activity	Bacteria (1 Eukaryotes/Metazoa + 1 Lentisphaerae)
**GSBLHT00006355001**	**5**	**IPR002198 SHORT-CHAIN DEHYDROGENASE/REDUCTASE SDR, IPR016040**	**oxidoreductase activity**	**Bacteria**
GSBLHT00002439001	6	IPR001279	hydrolase activity	Bacteria
GSBLHT00006858001	6	IPR001303	metal ion binding	Bacteria
GSBLHT00006397001	3	IPR002582,IPR004568,IPR008278	macromolecule biosynthetic process	Bacteria (3 Eukaryotes/Stramenopiles)
GSBLHT00004311001	6	IPR006992	metabolic process	Bacteroidetes (Bacteroidetes + 1 Firmicutes)
**GSBLHT00002943001 MFS Ortholog**	**5**	**IPR005829,IPR007114,IPR011701,IPR016196**	**integral to membrane**	**Bacteria (Eukaryotes/Alveolata,Fungi)**
GSBLHT00001574001	1	IPR002068,IPR008978		Bacteria

**GSBLHT00000637001 MFS Ortholog**	**5**	**IPR005829,IPR007114,IPR011701,IPR016196**	**integral to membrane**	**Bacteria (Eukaryotes/Alveolata,Fungi)**
**GSBLHT00004711001**	**1**	**IPR008009,IPR015919**	**Membrane**	**Homologues are too divergent, but likely bacterial**
GSBLHT00004237001	1	IPR001296	biosynthetic process	Firmicutes/Epsilonproteobacteria
**GSBLHT00001348001**	**1**	**IPR008009,IPR015919**	**Membrane**	**Homologues are too divergent, but likely bacterial**
GSBLHT00000893001	1	IPR002140	-	Eukaryotes/Archaea
GSBLHT00000081001	1	IPR011063,IPR012094	tRNA processing	Bacteria (4 eukaryotes + bacteria)
GSBLHT00006932001	4	IPR003141,IPR004013,IPR016195	DNA replication	Firmicutes
GSBLHT00004911001	7	IPR000825	iron-sulfur cluster assembly	Archaea
**GSBLHT00004498001**	**4**	**IPR000683 OXIDOREDUCTASE, N-TERMINAL, IPR004104,IPR016040**	**oxidoreductase activity**	**Bacteria**
GSBLHT00004455001	7	IPR007114,IPR011701,IPR016196	integral to membrane	Bacteria (1 Gammaproteobacteria)
GSBLHT00004383001	3	IPR002523	metal ion transmembrane transporter activity	Bacteria (2 archaea)
**GSBLHT00004202001**	**1**	**IPR008009,IPR008979,IPR015919**	**Membrane**	**Homologues are too divergent, but likely bacterial**
**GSBLHT00003545001**	**2**	**IPR008009,IPR015919**	**Membrane**	**Homologues are too divergent, but likely bacterial**
**GSBLHT00001822001 Lipase ortholog**	**1**	**IPR013094**	**hydrolase activity**	**Bacteria (various bacteria + 1 fungi)**
**GSBLHT00001821001**	**2**	**IPR008009,IPR008979,IPR011658,IPR015919**	**Membrane**	**Homologues are too divergent, but likely bacterial**
**GSBLHT00001768001**	**1**	**IPR008009,IPR015919**	**Membrane**	**Homologues are too divergent, but likely bacterial**
**GSBLHT00000696001**	**1**	**IPR008009,IPR015919**	**Membrane**	**Homologues are too divergent, but**

				**likely bacterial**
GSBLHT00007192001	2	-	-	Few eukaryotic homologues
GSBLHT00007018001	3	IPR009000	-	Bacteria (7 Eukaryotes/Fungi,Stramenopiles,Alveolata)
**GSBLHT00004337001**	**1**	**IPR008009**	**-**	**Homologues are too divergent, but likely bacterial**
**GSBLHT00004094001**	**1**	**IPR008009,IPR008979,IPR012633,IPR015919**	**Membrane**	**Homologues are too divergent, but likely bacterial**
**GSBLHT00003577001**	**1**	**IPR008009,IPR015919**	**Membrane**	**Homologues are too divergent, but likely bacterial**
GSBLHT00002916001	1	-	-	Firmicutes
**GSBLHT00002796001**	**1**	**IPR008009,IPR008979,IPR012633,IPR015919**	**Membrane**	**Homologues are too divergent, but likely bacterial**
**GSBLHT00002616001**	**1**	**IPR008009,IPR008979,IPR015919**	**Membrane**	**Homologues are too divergent, but likely bacterial**
GSBLHT00002595001	6	IPR003409	-	Bacteria/Eukaryotes
**GSBLHT00002408001**	**2**	**IPR008009,IPR008979,IPR015919**	**Membrane**	**Homologues are too divergent, but likely bacterial**
**GSBLHT00001785001**	**3**	**IPR002198 SHORT-CHAIN DEHYDROGENASE/REDUCTASE SDR, IPR016040**	**oxidoreductase activity**	**Bacteria (various bacteria + 1 fungi)**
GSBLHT00001681001	3	IPR004547,IPR006148	N-acetylglucosamine metabolic process	Bacterial/Fungi
**GSBLHT00001219001**	**1**	**IPR008009,IPR015919**	**Membrane**	**Homologues are too divergent, but likely bacterial**
GSBLHT00000566001	1	IPR001023,IPR013126	ATP binding	Bacteria (various bacteria + 1 archaea + 1 Eukaryote/Microsporidia)
GSBLHT00000458001	5	IPR000415	oxidoreductase activity	Archaea/Thaurmarchaeota
GSBLHT00000378001	8	IPR002786	-	Unknown (7 eukaryotes, 3 Bacteroidetes)

GSBLHT00006951001	5	-	-	Unknown (Homologues are too divergent)
GSBLHT00006644001	1	IPR001023,IPR013126	ATP binding	Bacteria (1 Eukaryotes/Parabasalia)
GSBLHT00006445001	4	-	-	Bacteria likely Firmicutes
GSBLHT00006416001	3	IPR013216	methyltransferase activity	Bacteria (1 Chloroflexi)
GSBLHT00006169001	9	-	-	Firmicutes, Actinobacteria, Eukaryotes/Euglenozoa
GSBLHT00006124001	6	IPR000086,IPR015797	hydrolase activity	Eukaryotes/Epsilonproteobacteria
GSBLHT00005059001	1	IPR002528,IPR015522	Membrane	Bacteria likely Firmicutes
**GSBLHT00004659001**	**1**	**IPR008009,IPR008979,IPR015919**	**Membrane**	**Homologues are too divergent, but likely bacterial**
GSBLHT00004487001	2	IPR001173	-	Bacteria likely Firmicutes
GSBLHT00004436001	2	IPR000177,IPR003014,IPR006626,IPR011050	blood coagulation	Bacteria likely Bacteroidetes
GSBLHT00004369001	6	IPR000600	-	Bacteroidetes/Actinobacteria (1 Eukaryotes/Stramenopiles)
GSBLHT00004169001	2	-	-	Eukaryotes (few alphaproteobacteria)
**GSBLHT00003996001**	**1**	**IPR008009,IPR008979,IPR015919**	**Membrane**	**Homologues are too divergent, but likely bacterial**
**GSBLHT00003766001**	**1**	**IPR008009**	**-**	**Homologues are too divergent, but likely bacterial**
GSBLHT00003763001	1	IPR001104,IPR010721	integral to membrane	Bacteria/Eukaryotes likely Eukaryotes
GSBLHT00003361001	2	IPR011611	-	Bacteria (1 Eukaryotes/Green algae)
**GSBLHT00003301001**	**1**	**IPR008009,IPR015919**	**Membrane**	**Homologues are too divergent, but likely bacterial**
GSBLHT00003198001	1	IPR000600	-	Bacteria (1 Actinobacteria)
**GSBLHT00002601001**	**1**	**IPR008009,IPR008979,IPR015919**	**Membrane**	**Homologues are too divergent, but likely bacterial**
GSBLHT00002502001	6	IPR013217	-	Eukaryotes/Bacteria
GSBLHT00002409001	1	IPR002528,IPR015522	Membrane	Bacteria

GSBLHT00002360001	6	IPR000415	oxidoreductase activity	Bacteria
**GSBLHT00002149001**	**1**	**IPR008009,IPR008979,IPR015919**	**Membrane**	**Homologues are too divergent, but likely bacterial**
**GSBLHT00002146001**	**1**	**IPR008009,IPR008979,IPR015919**	**Membrane**	**Homologues are too divergent, but likely bacterial**
**GSBLHT00002134001**	**5**	**IPR000421,IPR008009,IPR008979,IPR015919**	**Membrane**	**Homologues are too divergent, but likely bacterial**
GSBLHT00002047001	6	IPR007392	hydro-lyase activity	Bacteria
GSBLHT00002010001	1	IPR000182,IPR016181	metabolic process	Bacteria likely Firmicutes
**GSBLHT00001912001 MFS Ortholog**	**2**	**IPR005828,IPR005829,IPR007114,IPR016196**	**integral to membrane**	**Bacteria (Eukaryotes/Alveolata,Fungi)**
**GSBLHT00001890001**	**2**	**IPR008009**	**-**	**Homologues are too divergent, but likely bacterial**
GSBLHT00001632001	1	IPR006637	-	Bacteria (Firmicutes + Actinobacteria)
GSBLHT00001425001	1	IPR003409,IPR011652		Eukaryotes/Bacteria
**GSBLHT00001307001**	**1**	**IPR008009,IPR008979,IPR015919**	**Membrane**	**Homologues are too divergent, but likely bacterial**
GSBLHT00001094001	4	-	-	Proteobacteria (6 betaproteobacteria + 7 deltaproteobacteria)
GSBLHT00000959001	2	IPR011008	-	Bacteria (various bacteria + 2 alveolata)
GSBLHT00000930001	1	IPR002068,IPR008978	-	Bacteria (2 Actinobacteria + 3 3 Eukaryotes)
GSBLHT00000920001	5	-	-	Bacteroidetes
**GSBLHT00000465001**	**1**	**IPR008009,IPR008979,IPR015919**	**Membrane**	**Homologues are too divergent, but likely bacterial**
GSBLHT00000353001	2	-	-	No homologues detected
GSBLHT00000160001	2	-	-	Bacteria (3 eukaryotes, 2 bacteria)
GSBLHT00002116001	4	IPR002942,IPR006145	Pseudouridine synthase activity	Eukaryotes/Alphaproteobacteria
GSBLHT00003001001	4	IPR000449,IPR001816,IP	translational elongation	Bacteria

		R009060,IPR014039		
GSBLHT00002454001	1	IPR004770	integral to membrane	Bacteria likely Firmicutes
GSBLHT00004726001	3	IPR004770	integral to membrane	Bacteria
GSBLHT00002645001	1	IPR004770	integral to membrane	Bacteria/Archaea likely Firmicutes
GSBLHT00000336001	5	IPR001926	pyridoxal phosphate binding	Bacteria/Eukaryotes (1 Spirochaetes and Eukaryotes)
GSBLHT00002170001	1	IPR004770	integral to membrane	Bacteria (1 Eukaryotes/Stramenopiles + various bacteria)
GSBLHT00005048001	1	IPR004770	integral to membrane	Bacteria
GSBLHT00006555001	9	IPR000209,IPR015500	Proteolysis	Eukaryotes/Bacteria
GSBLHT00003375001	6	IPR002314,IPR006195,IPR015805	histidyl-tRNA aminoacylation	Bacteria likely alphaproteobacteria (1 Eukaryotes/Amoebozoa)
GSBLHT00006366001	5	-	-	Bacteria
GSBLHT00001906001	2	IPR002772	carbohydrate metabolic process	Bacteria/Bacteroidetes
GSBLHT00006363001	6	-	-	Bacteria (1 Eukaryotes/Fungi)
GSBLHT00003854001	6	IPR006597,IPR011990	Binding	Bacteria (1 Betaproteobacteria + 3 Eukaryotes/Stramenopiles,Amoebozoa)
GSBLHT00001500001	7	IPR001327,IPR001763,IPR004099,IPR013027,IPR016156	FAD binding	Bacteria (4 proteobacteria + 1 green algae)
GSBLHT00002430001	4	IPR007835	-	Bacteria (various bacteria + S Eukaryotes/Stramenopiles)
GSBLHT00000166001	9	IPR001327,IPR001763,IPR004099,IPR013027,IPR016156	FAD binding	Bacteria (4 bacteria, 1green algae)
GSBLHT00001833001	3	-	-	Bacteria/Eukaryotes (Proteobacteria + Spirochaetes + Stramenopiles)
GSBLHT00004516001	9	IPR005000,IPR015813	carbon-carbon lyase activity	Bacteria likely Proteobacteria

Table S4. Predicted secretory proteins of *Blastocystis sp.* subtype 7

**Locus Genome Browser**	**Name**
	**Cytosol stress response/chaperones**
GSBLHT00000633001	HSP
GSBLHT00000826001	Chaperone GrpE
GSBLHT00002581001	chaperone protein DnaJ [*Rhodobacter sphaeroides* ATCC 17029]
GSBLHT00007172001	hypothetical protein, conserved [*Toxoplasma gondii* ME49]
GSBLHT00003792001	DNAJ homolog subfamily A member 1 [*Entamoeba histolytica* HM-1:IMSS]
GSBLHT00005138001	heat shock protein [*Thalassiosira pseudonana* CCMP1335]
	**Cytosol energy metabolism and glycoside hydrolase**
GSBLHT00006907001	Ferredoxin, 2Fe-2S [*Tetrahymena thermophila* SB210]
GSBLHT00000617001	Phosphoenolpyruvate synthase
GSBLHT00001190001	ADP-ribosylglycohydrolase
GSBLHT00001905001	IPR017853 Glycoside hydrolase
GSBLHT00000302001	O-glycosyl hydrolase
GSBLHT00000713001	β-galactosidase
GSBLHT00000838001	β-galactosidase
GSBLHT00002785001	similar to galactosidase, beta 1 [*Hydra magnipapillata*]
GSBLHT00003656001	putative beta-galactosidase [*Arabidopsis thaliana*]
GSBLHT00004414001	mannose-6-phosphate protein p76 [*Homo sapiens*]
GSBLHT00003872001	enoyl Coenzyme A hydratase domain containing 1 isoform 2 [*Bos taurus*]
GSBLHT00004751001	similar to lysosomal beta-galactosidase [*Strongylocentrotus purpuratus*]
	**Structural/cytoskeletal**
GSBLHT00006206001	capsular polysaccharide biosynthesis protein-like [*Trichodesmium*]
	**Proteases**
GSBLHT00001111001	Peptidase C13, **legumain**

GSBLHT00001253001	Cysteine protease, **cathepsin L**
GSBLHT00001036001	Cysteine protease, **cathepsin L**
GSBLHT00001922001	Putative cysteine protease (peptidase C13 family, **legumain**)
GSBLHT00002757001	Peptidase C13 family protein, **legumain** [*Tetrahymena thermophila* SB210]
GSBLHT00002773001	cysteine protease [*Phytophthora infestans*]
GSBLHT00002821001	**cathepsin B** [*Trichobilharzia szidati*]
GSBLHT00002848001	Peptidase C13 family protein, **legumain** [*Tetrahymena thermophila* SB210]
GSBLHT00002373001	cathepsin-like cysteine protease, **cathepsin C** [*Phytophthora infestans*]
GSBLHT00002507001	Peptidase C1 subfamily
GSBLHT00004523001	putative cysteine protease, **cathepsin L** [*Sorogena stoianovitchae*]
GSBLHT00003973001	subtilisin-like serine protease-like [*Pelodictyon luteolum* DSM]
GSBLHT00005004001	cathepsin-like cysteine protease, **cathepsin C** [*Phytophthora infestans*]
GSBLHT00005100001	**cathepsin B** [*Paralichthys olivaceus*]
GSBLHT00005101001	**cathepsin B**
GSBLHT00006028001	Papain family cysteine protease [*Tetrahymena thermophila* SB210]
GSBLHT00004670001	cysteine protease, **legumain** [*Phytophthora infestans*]
GSBLHT00006555001	peptidase S8 and S53 subtilisin kexin sedolisin [*Geobacter* sp.]
	**Hexose-containing molecule digestion**
GSBLHT00000292001	Hexosaminidase superfamily
GSBLHT00002531001	alpha-L-fucosidase [*Capnocytophaga sputigena* Capno]
GSBLHT00004919001	QRT3 (QUARTET 3, polygalacturonase) [*Arabidopsis thaliana*]
	**Protein folding**
GSBLHT00000107001	Cyclophilin
GSBLHT00002105001	Thioredoxin-like superfamily, disulfide isomerase [*Entamoeba dispar* SAW760]
GSBLHT00002808001	protein disulfide isomerase [*Haliotis discus discus*]
GSBLHT00002347001	putative disulphide isomerase [*Ascaris suum*]
GSBLHT00002631001	Cyclophilin

GSBLHT00003561001	PDIL2-2 - Zea mays protein disulfide isomerase
GSBLHT00004589001	cyclophilin [*Bigelowiella natans*]
GSBLHT00006025001	disulfide isomerase [*Culex quinquefasciatus*]
GSBLHT00006471001	protein disulfide isomerase-like protein [*Glycine max*]
GSBLHT00006585001	protein disulfide isomerase, isoform A [*Drosophila melanogaster*]
	**Glycosyltransferases**
GSBLHT00000162001	Trefoil domain protein
GSBLHT00001093001	Glycosyltransferase
GSBLHT00001007001	Glycosyltransferase
GSBLHT00003670001	glycosyl transferase [*Lyngbya* sp. PCC 8106]
GSBLHT00004487001	glycosyl transferase family protein [*Methylobacterium radiotolerans*]
GSBLHT00003979001	Chain A of fucosyltransferase Nodz From *Bradyrhizobium*
GSBLHT00004049001	dolichyl-diphosphooligosaccharide-protein glycotransferase [*Bombyx mori*]
GSBLHT00006287001	UDP-Gal:betaGlcNAc beta 1,3-galactosyltransferase polypeptide
GSBLHT00006751001	carbohydrate binding [*Arabidopsis thaliana*], nodal modulator
GSBLHT00006556001	N-acetylglucosamine-1-phosphottransferase, gamma subunit, putative
	**Protease inhibitors**
GSBLHT00001994001	type I proteinase inhibitor-like protein [*Citrus paradisi*] chymotrypsin
GSBLHT00002799001	cystatin A [*Mus musculus*]
GSBLHT00005077001	similar to endopeptidase inhibitor-like [*Tribolium castaneum*]
	**Protein binding**
GSBLHT00006030001	Immunoglobulin heavy chain-binding protein homolog
GSBLHT00006428001	CS domain-containing protein [*Cryptosporidium muris* RN66]
	**Transporters**
GSBLHT00002158001	phosphatidylglycerol/phosphatidylinositol transfer protein

GSBLHT00004624001	Phosphatidylglycerol/phosphatidylinositol transfer protein
	**Penicillin**
GSBLHT00004835001	Acyl-coenzyme A:6-aminopenicillanic acid acyl-transferase
	Other functions
GSBLHT00000772001	Interferon γ inducible thiol reductase
GSBLHT00002755001	similar to lysosomal thiol reductase IP30 precursor [*Tribolium castaneum*]
GSBLHT00000297001	G-protein coupled receptor (GPCR)
GSBLHT00000765001	Interferon γ inducible protein
GSBLHT00003782001	protein kinase C substrate 80K-H [*Xenopus laevis*]
GSBLHT00003295001	serine threonine phosphatase [*Thalassiosira pseudonana* CCMP1335]
GSBLHT00003932001	Ras family protein [*Tetrahymena thermophila* SB210]

Table S5. ***Blastocystis sp.*** **subtype 7 encoding potential antioxidant enzymes and proteins involved in glutathione synthesis.**

Gene name	Locus Genome Browser	BLASTP best hit	Predicted localization
Dimeric iron-containing superoxide dismutase (SOD1)	GSBLHT00002972001	*Burkholderia dolosa* (superoxide dismutase)	cytosolic
Dimeric iron-containing superoxide dismutase (SOD2)	GSBLHT00000584001	*Methylophaga tiooxidans* (superoxide dismutase)	MLO
Thioredoxin (WCAPC and WCGPC motifs)	GSBLHT00004503001	*Picea sitchensis* (unknown)	cytosolic
Thioredoxin (WCRPC motif)	GSBLHT00003598001	*Micromonas* sp. (predicted protein)	MLO
Thioredoxin reductase (selenoprotein type H-TrxR)	GSBLHT00004575001	*Monosiga brevicollis* (unknown)	cytosolic
Thioredoxin reductase (selenoprotein type H-TrxR)	GSBLHT00003465001	*Monosiga brevicollis* (unknown)	cytosolic
Thioredoxin reductase (nonselenium H-TrxR)	GSBLHT00002152001	*Xenopus laevis* (thioredoxin reductase)	cytosolic
2-Cys peroxiredoxin (typical 2-Cys Prx)	GSBLHT00001088001	*Tetrahymena thermophila* (AhpC/TSA family protein)	cytosolic
2-Cys peroxiredoxin (typical 2-Cys Prx)	GSBLHT00001271001	*Tetrahymena thermophila* (AhpC/TSA family protein)	cytosolic
2-Cys peroxiredoxin (typical 2-Cys Prx)	GSBLHT00001529001	*Tetrahymena thermophila* (AhpC/TSA family	cytosolic

		protein)	
2-Cys peroxiredoxin (typical 2-Cys Prx)	GSBLHT00001960001	*Tetrahymena thermophila* (AhpC/TSA family protein)	cytosolic
2-Cys peroxiredoxin (typical 2-Cys Prx)	GSBLHT00001970001	*Tetrahymena thermophila* (AhpC/TSA family protein)	cytosolic
2-Cys peroxiredoxin (typical 2-Cys Prx)	GSBLHT00002298001	*Tetrahymena thermophila* (AhpC/TSA family protein)	cytosolic
